# The mechanosensitive channel Piezo1 cooperates with semaphorins to control neural crest migration

**DOI:** 10.1242/dev.200001

**Published:** 2021-12-02

**Authors:** Brenda Canales Coutiño, Roberto Mayor

**Affiliations:** Department of Cell and Developmental Biology, University College London, Gower Street, London WC1E 6BT, UK

**Keywords:** Cell migration, Mechanosensing, Piezo1, Sema, Rac1, Neural crest, *Xenopus*

## Abstract

Cells are permanently exposed to a multitude of different kinds of signals: however, how cells respond to simultaneous extracellular signals within a complex *in vivo* environment is poorly understood. Here, we studied the role of the mechanosensitive ion channel Piezo1 on the migration of the neural crest, a multipotent embryonic cell population. We identify that Piezo1 is required for the migration of *Xenopus* cephalic neural crest. We show that loss of Piezo1 promotes focal adhesion turnover and cytoskeletal dynamics by controlling Rac1 activity, leading to increased speed of migration. Moreover, overactivation of Rac1, due to Piezo1 inhibition, counteracts cell migration inhibitory signals by Semaphorin 3A and Semaphorin 3F, generating aberrant neural crest invasion *in vivo*. Thus, we find that, for directional migration *in vivo*, neural crest cells require a tight regulation of Rac1, by semaphorins and Piezo1. We reveal here that a balance between a myriad of signals through Rac1 dictates cell migration *in vivo*, a mechanism that is likely to be conserved in other cell migration processes.

## Abstract (precis)

**Summary:** Neural crest directional and normal migration *in vivo* requires both chemical and mechanical regulation of Rac1 by Semaphorin 3A and Piezo1, respectively.

## INTRODUCTION

Directional cell migration is orchestrated by an interplay of extracellular and intrinsic signals. Failure in cell migration leads to defects in embryonic development, wound healing and immune response, and cell migration is also involved in cancer invasion during tumour progression (Mayor and Etienne-Manneville, 2016; Yamada and Sixt, 2019). The complex behaviour of migratory cells can be observed in neural crest (NC) cells, which are a multipotent embryonic cell population programmed to migrate long distances within the embryo in a collective and directional manner (Szabó and Mayor, 2018).

NC cells detect and respond to different extracellular stimuli to shape their migratory path. Gradients of chemoattractants, such as Sdf1 (also known as Cxcl12), are expressed along the direction of NC migration and promote protrusion formation via the activation of the small GTPase Rac1 (Theveneau et al., 2010; Shellard et al., 2018). Protrusion formation facilitates cell migration via traction forces generated from focal adhesion (FA) proteins, which connect the actin cytoskeleton to the extracellular matrix at the protrusion sites via integrins (Seetharaman and Etienne-Manneville, 2020). The chemoattractant signals are competed *in vivo* by migration repellents, such as semaphorins (Sema), ephrins and Slit/Robo (Theveneau and Mayor, 2012), which are expressed at the borders of the NC and prevent the invasion of the NC cells to nearby tissues by inhibiting Rac1 activity, which leads to protrusion collapse (Bajanca et al., 2019). However, NC migration can follow an organised pattern even in the absence of Sema-repellent signals (Bajanca et al., 2019), indicating that additional unidentified mechanisms could regulate and prevent NC invasion to nearby tissues. Moreover, how cells are able to integrate simultaneous extracellular signals in the complexity of the 3D environment *in vivo* remains poorly understood.

NC and other migratory cells are now known to express different mechanosensitive channels (Simões-Costa et al., 2014), including mechanosensitive (MS) ion channels, such as Piezo1. However, the role of Piezo1 in cell migration *in vivo* is unclear. Piezo1 is a transmembrane ion channel, exclusively activated by mechanical forces in biological systems (Coste et al., 2010; Lin et al., 2019). Upon Piezo1 activation, calcium ions enter the cell triggering a biochemical cascade, and thereby linking mechanical forces with complex biochemical signalling (Canales Coutiño and Mayor, 2021). Here, we identify Piezo1 as an important MS channel in *Xenopus* migratory NC cells. We show that Piezo1 controls Rac1 and FA dynamics *in vitro*, and that these activities are essential to counterbalance the inhibitory signals Sema3A and Sema3F during NC migration *in vivo*. Here, we reveal that cooperative regulation of Rac1 by semaphorins and Piezo1 dictates cell migration *in vivo*.

## RESULTS

### Piezo1 inhibition increases speed of neural crest cell dispersion

**Fig. 1.**
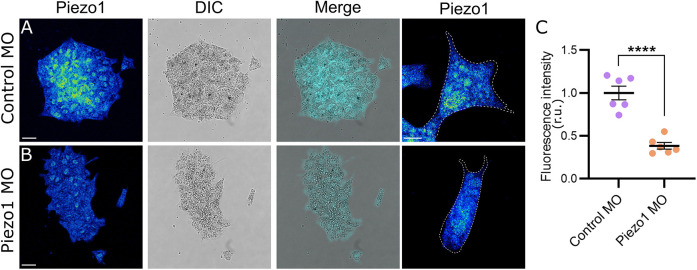
**Piezo1 is expressed in migratory neural crest cells.** (A,B) *Xenopus* NC explants plated on a fibronectin-coated dish and immunostained against Piezo1 in control MO (A) and Piezo1 MO (B). Scale bars: 20 μm. (C) Quantification of fluorescent intensity of Piezo1 from A,B. r.u., relative units. *n*=60 cells per condition. Error bars are ±s.e.m. Each dot is the mean value of an independent experiment. All data are representative of at least three biological replicates. ****P≤0.0001 (unpaired two-tailed Student's *t*-test).

**Fig. 2.**
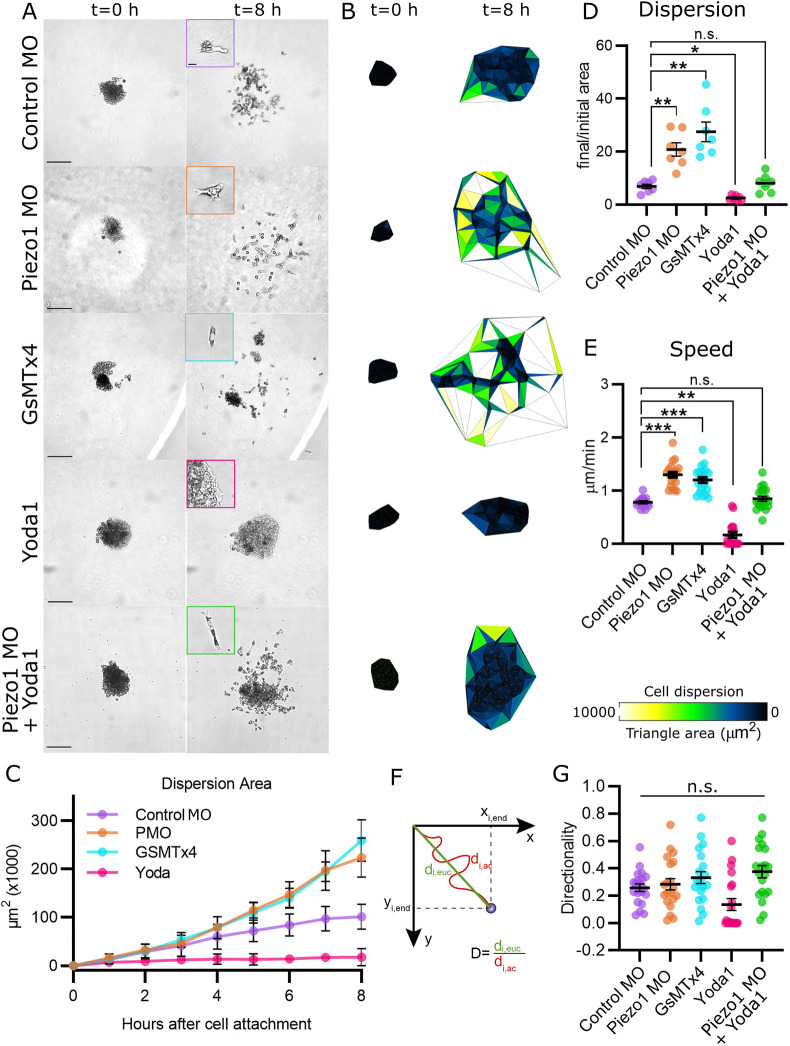
**Piezo1 inhibition increases speed of neural crest cell dispersion.** (A) Representative images of NC explants plated on top of fibronectin at time 0 (left column) and after 8 h (right column). Note that genetic (Piezo1 MO) and chemical (GsMTx4) inhibition of Piezo1 increases cell dispersion. The Piezo1 agonist (Yoda1) inhibits cell dispersion. Yoda1 treatment on Piezo1 MO explants rescues the dispersion phenotype to control levels. Scale bars: 20 μm. (B) Analysis of cell dispersion area by Delaunay triangulation, representative areas from A. (C) Quantification of hourly area of cell dispersion from A. *n*=35 explants per condition. (D) Normalised area of cell dispersion, showing a ratio of final and initial area from A. *n*=35 explants per condition. (E) Quantification of speed of cell migration from A. (F) Illustration of how directionality is calculated. The Euclidean distance (d_i.euc_, ideal distance following a straight path) is divided by the accumulated distance (d_i.ac_, real distance the cell travelled). Cells with values closer to 1 migrated in a more directional manner. (G) Quantification of directionality of cell migration from A. (D-G) *n*=60 cells per condition. Error bars are ±s.e.m. Each dot is the mean value of an independent experiment. All data are representative of at least three biological replicates. ***P≤0.001, **P≤0.01, *P≤0.05 (one-way ANOVA with a Dunnett's multiple comparisons post-test). n.s., non-significant.

Consistent with previous RNA-seq studies (Simões-Costa et al., 2014), we showed that the Piezo1 protein is expressed in migratory NC (Fig. 1A). Piezo1 levels can be efficiently knocked down (Fig. 1B,C) by expressing a previously characterized Piezo1 antisense morpholino (Piezo1 MO) (Koser et al., 2016). To study the role of Piezo1 in NC migration, we performed time-lapse microscopy of NC explants cultured on fibronectin (Fig. 2A; Movie 1). Control NC explants spread and migrate as single cells as previously described (Bahm et al., 2017). Surprisingly, Piezo1 MO increased cell dispersion compared with control cells (Fig. 2A-D). To support the specificity of this phenotype we proceeded to use the chemical inhibitor of Piezo1 (GsMTx4; Bae et al., 2011) and a Piezo1 activator (Yoda1; Botello-Smith et al., 2019). Treatment of NC cells with the Piezo1 inhibitor GsMTx4 promoted cell dispersion similar to Piezo1 morphant cells, whereas the Piezo1 activator Yoda1 inhibited cell dispersion (Fig. 2A-D). Moreover, we rescued the Piezo1 MO phenotype by treating neural crest cells with the Piezo1 activator Yoda1, leading to cell dispersion equivalent to control levels (Fig. 2A,B,D), demonstrating the specificity of the Piezo1 MO. Finally, we verified the efficiency and reversibility of the drugs by measuring calcium levels (Fig. S2A,B) and demonstrate that Piezo1 MO does not induce cell death (Fig. S1A,B). In conclusion, inhibition of Piezo1 promotes cell dispersion *in vitro*.

To understand the mechanism by which a Piezo1 inhibition increases dispersion of NC explants, single cell motility parameters were quantified. Speed of cell migration was increased after genetic and chemical inhibition of Piezo1, whereas Piezo1 activation by Yoda1 decreased speed of cell migration (Fig. 2E). Speed of migration in Piezo1 MO cells was rescued by the Piezo1 activator Yoda (Fig. 2E). In addition, there was no change in directionality among all the conditions studied (Fig. 2F,G). Moreover, our hourly analysis of cell dispersion indicates that the time of cell dissociation is not affected by Piezo1 loss-of-function (Fig. 2C, hours 0-4), which suggests that cell-cell adhesion is not affected. Taken together, these results show that Piezo1 inhibition in the NC increases the speed of cell migration.

### Increased FA turnover in Piezo1 knockdown

**Fig. 3.**
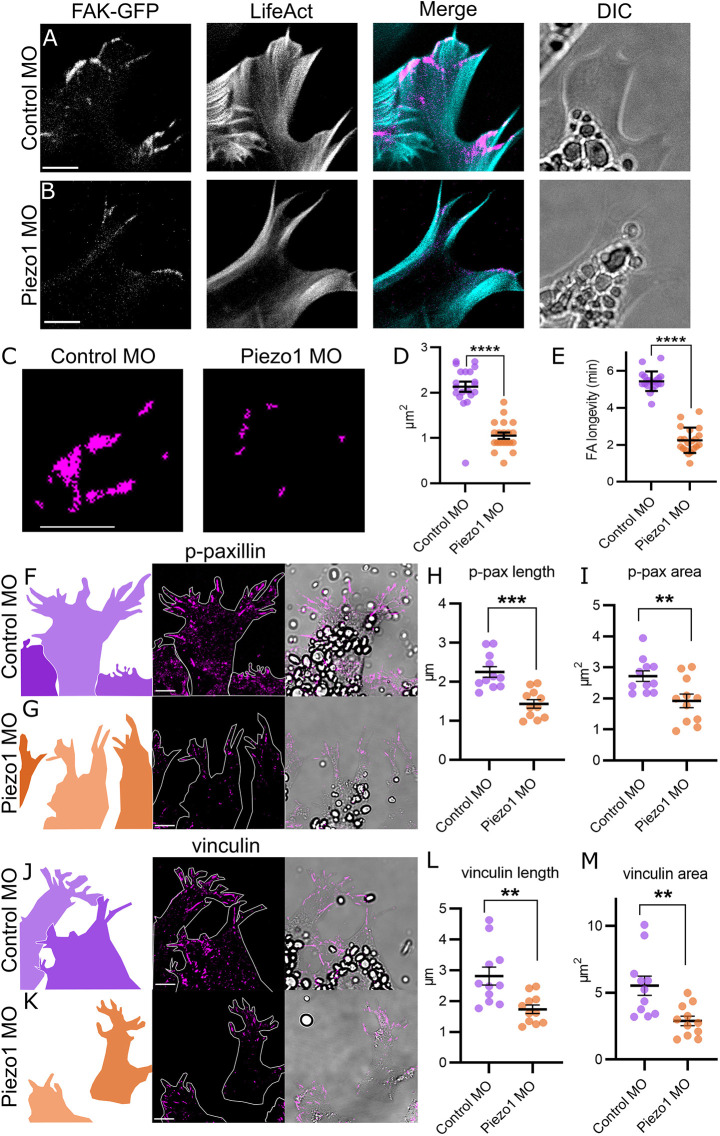
**Focal adhesion regulation by Piezo1.** (A,B) Cell protrusions from NC cells plated on fibronectin and expressing focal adhesion kinase tagged with GFP (FAK-GFP) and LifeAct-Ruby in control MO (A) and Piezo1 MO (B). (C) Representative masks of the FAK-GFP from A,B. (D) Quantification of area of FAK-GFP normalised to control from C. (E) Quantification of focal adhesion longevity in minutes from A,B. *n*=20 cells in each condition. (F,G,J,K) *Xenopus* NC explants plated on a fibronectin-coated dish. Cell shapes, cell contours and brightfield images are shown in left, middle and right columns, respectively. Explants immunostained against p-paxillin (F,G) or vinculin (J,K) in control MO (F,J) and Piezo1 MO (G,K). (H,I) Quantification of length (H) and area (I) of individual focal adhesions from F,G. (L,M) Quantification of length (L) and area (M) of individual focal adhesions from J,K. Note that focal adhesions are shorter and smaller in Piezo1 MO compared with control. *n*=110 cells of 11 explants in each condition. Each dot is the mean value of an independent experiment, representing the average of all FAs measured within an explant (all the FA of ten cells were measured per explant). All data are representative of at least three biological replicates. Error bars are ±s.e.m. ****P≤0.0001, ***P≤0.001, **P≤0.01 (unpaired two-tailed Student's *t*-test). Scale bars: 5 µm.

To identify the mechanism by which Piezo1 inhibition increased the speed of cell migration we analysed FAs. FA proteins, which link the extracellular matrix to the actin cytoskeleton, are required for cell-matrix adhesion, mechanotransduction and cytoskeletal organization (Parsons et al., 2010; Barriga et al., 2018). Dynamic FA regulation, characterized by smaller FAs and increased turnover, leads to increased speed of mesenchymal cell migration (Kim and Wirtz, 2013; Hu et al., 2017). Moreover, higher speed of cell migration upon Piezo1 inhibition has been directly linked to increased FA turnover in breast cancer cells (Yu et al., 2021). To characterize the effect of Piezo1 inhibition on FA, we directly visualised the dynamics of FAs during cell migration, we expressed Focal Adhesion Kinase tagged with GFP (FAK-GFP) in migrating NC cells, and time-lapse video-microscopy was performed at a high spatiotemporal resolution (Fig. 3A,B). FAK-GFP was localized in typical FA domains along the actin protrusions in control NC migrating cells (Fig. 3A), whereas in Piezo1 MO cells, FAK-GFP signal was reduced (Fig. 3B). Quantification of the FAK-GFP area showed a significant decreased in Piezo1-depleted cells compared with control cells (Fig. 3C,D). Importantly, analysis of the stability of FAK-GFP during cell migration showed a clear decrease in FA longevity in Piezo1 knockdown (KD) cells compared with control (Fig. 3E).

In addition, we studied the localization of phospo-paxillin (p-paxillin) and vinculin, as previously described (Roycroft et al., 2018). Immunostaining of p-paxillin (Fig. 3F,G) and vinculin (Fig. 3J,K) was performed in NC explants of control and Piezo1 MO. A decrease in FA length and area was observed in Piezo1 MO compared with control explants, and in p-paxillin (Fig. 3H,I) and vinculin (Fig. 3L,M), consistent with the reduced area of FAK-GFP, previously described (Fig. 3D). These results suggest that Piezo1 MO cells have a more dynamic regulation of FA proteins, p-paxillin and vinculin, compared with control NC cells.

In summary, loss of Piezo1 in NC cells leads to a decrease in size, area and longevity of FAs. These results indicate a higher turnover and more dynamic FA regulation in Piezo1-depleted cells compared with control NC cells, which is consistent with an increased migration in cells lacking Piezo1.

### Loss of Piezo1 leads to increased protrusion activity via Rac1 activation

**Fig. 4.**
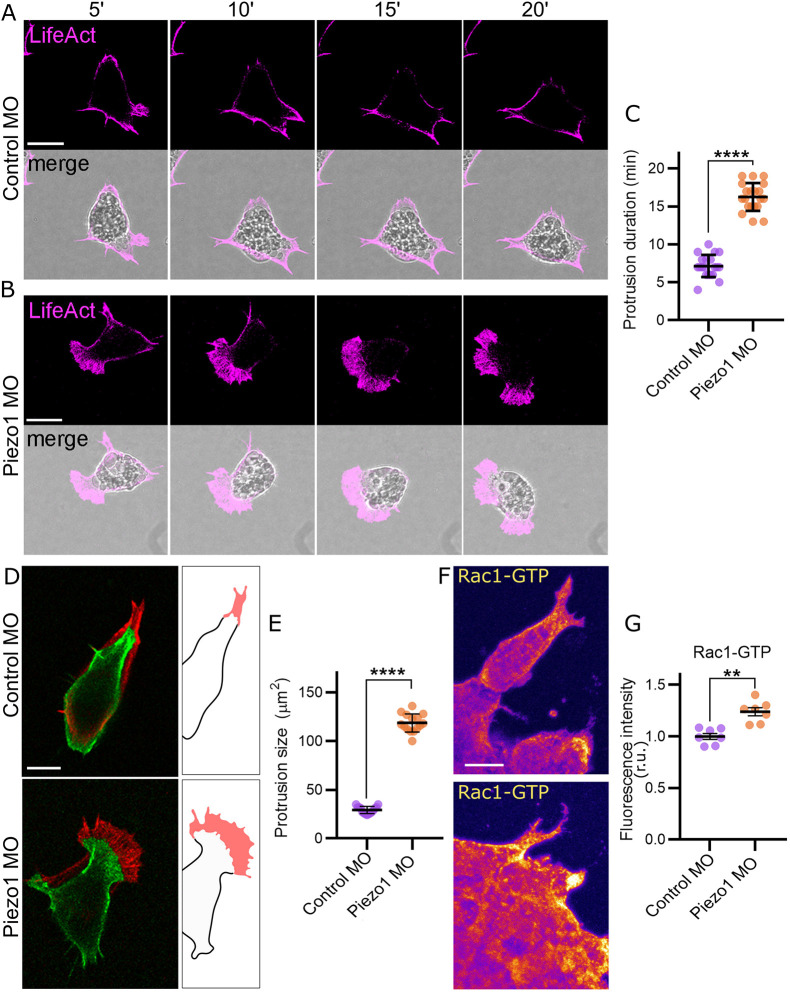
**Loss of Piezo1 leads to increased protrusion activity via Rac1 activation.** (A,B) NC cells plated on a fibronectin-coated dish, expressing LifeAct-Ruby in control MO (A) and Piezo1 MO (B) at different time points (top). Note that cell protrusions are more abundant in Piezo1 MO compared with control MO. (C) Quantification of the protrusion duration in minutes from A,B. *n*=18 cells in each condition. (D) Time projection of NC cells from A,B at time 0 (green) and after 7 min of migration (red). Protrusion area is highlighted in red in the illustration. (E) Quantification of the area of cell protrusions from A,B. *n*=18 cells in each condition. (F) Immunostaining of Rac1-GTP (active Rac1) in control MO NC explants (top) and Piezo1 MO (bottom). (G) Quantification of fluorescence intensity of Rac1-GTP from F. r.u., relative units. *n*=70 cells in each condition. Each dot is the mean value of an independent experiment. All data are representative of at least three biological replicates. Error bars are ±s.e.m. **P≤0.01, ****P≤0.0001 (unpaired two-tailed Student's *t*-test). Scale bars: 10 µm.

Speed of cell migration and FA regulation are tightly linked to the actin cytoskeleton dynamics (Parsons et al., 2010). To determine the effect of Piezo1 upon the actin cytoskeleton organization during NC migration, we expressed LifeAct-Ruby alone and in the presence of the Piezo1 MO and analysed the actin protrusions during cell migration (Fig. 4A,B; Movie 2). We found a 3-fold increase in the duration of actin-based protrusions in Piezo1 MO compared with control cells (Fig. 4C). In addition, the area of protrusions increased significantly in Piezo1 MO compared with control (Fig. 4D,E). Overall, these results indicate an increase in protrusion formation and stability in Piezo1 MO.

The formation and stability of actin-based protrusions are regulated by the small GTPase Rac1 (Matthews et al., 2008). To determine whether loss of Piezo1 affects active Rac1 levels, we investigated Rac1 activity within migratory NC explants by immunostaining of Rac1-GTP – this antibody recognizes specifically the active form of Rac1 (Rac1 GTP-bound) (Cao et al., 2015) (Fig. 4F). We found that active Rac1 levels are significantly increased in Piezo1 MO compared with control NC cells (Fig. 4G). Taken together, these results suggest that loss of Piezo1 increases Rac1 activity, which in turn promotes cytoskeletal dynamics and protrusion formation.

### Loss of Piezo1 leads to NC migration defects *in vivo*

**Fig. 5.**
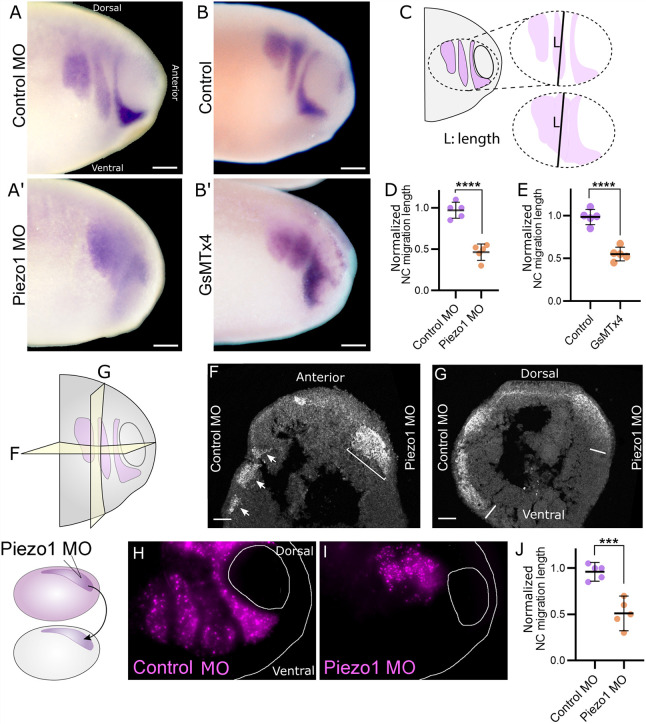
**Loss of Piezo1 leads to neural crest migration defects *in vivo*.** (A-B′) Whole-embryo *in-situ* hybridisation against the NC marker *Twist1* in control MO (A), Piezo1 MO (A′), control injected with 0.1× MMR (B) and treated with GsMTx4 (B′) embryos. (C) Schematic of a *Xenopus* embryo with NC streams colorized in purple, illustrating that the stream length was measured at the middle point of the total neural crest width in D,E,J. In control embryos this corresponds to the second stream (top), in Piezo1 MO stream definition is lost (bottom). (D,E) Quantification of the NC migration length as illustrated in C, from Piezo1 MO and its control MO shown in A,A′ (D) and GsMTx4 and its control shown in B,B′ (E). (F,G) Transverse (F) and sagittal (G) cryosections of whole embryos injected on the right side with Piezo1 MO and on the left side with control MO. The neural crest is labelled by fluorescence *in situ* hybridization against *Twist1*. (F) The three migratory NC streams can be observed on the control MO side (arrows) of transverse cryosections, stream definition is lost in the Piezo1 MO side (bracket). (G) Sagittal cryosections of whole embryos showing the second NC stream on the control MO side show the ventral migration of the NC cells (left line), the Piezo1 injected side is shorter (right line). (H,I) Non-fluorescent host embryos are grafted NC from donors expressing nuclear-RFP (left diagram). NC graft of control MO (H) and Piezo1 MO (I). (J) Quantification of the NC migration length from F,G. (D,E,J) *n*=5 embryos from each condition. Each dot is the mean value of an independent experiment. All data are representative of at least three biological replicates. Error bars are ±s.e.m. ***P≤0.001, ****P≤0.0001 (unpaired two-tailed Student's *t*-test). Scale bars: 100 µm.

Next, we investigated the effect of Piezo1 KD on NC migration during embryonic development *in vivo*. Piezo1 MO was injected into one half of the embryo at the eight-cell stage and *in situ* hybridization against the NC marker *Twist1* was performed. Severe defects in NC migration were observed in the Piezo1 MO-injected side compared with the control (Fig. 5A,A′). The well-defined migration pattern, in the form of three migratory NC streams, characteristic of NC migration (Fig. 5A), were often lost in the Piezo1 MO-injected side (Fig. 5A′). The same effect was observed when Piezo1 was inhibited chemically by GsMTx4 (Fig. 5B,B′). Upon quantification of the length of the NC migratory streams (Fig. 5C), we identified that NC streams are shorter in Piezo1 MO (Fig. 5D) and GsMTx4 (Fig. 5E) compared with control, indicating that Piezo1 is required for normal NC migration in *Xenopus in vivo*.

To better characterize the effect of Piezo1 KD on NC migration *in vivo*, transverse (Fig. 5F) and sagittal (Fig. 5G) embryo cryosections were analysed. Embryos were injected with the Piezo1 MO on the right side and with a control MO on the left side and fixed at migratory NC stages (stage 24). The NC was labelled by fluorescence *in situ* hybridization (FISH) against *Twist1*. Although the three migratory NC streams were easily identified on the control side of transverse cryosections, these streams appeared to be merged on the Piezo1 MO-injected side (Fig. 5F). Sagittal cryosections of the second NC stream on the control side show ventral migration of the NC cells, whereas the Piezo1 MO-injected side is shorter and the edges are less defined (Fig. 5G). Together, these observations indicate that Piezo1 is required for the stream migration of cephalic neural crest migration *in vivo*.

To determine whether the role of Piezo1 in NC migration *in vivo* is cell autonomous, we performed NC grafting experiments. NC injected with nuclear-RFP alone, or in combination with Piezo1 MO, were transplanted into uninjected host embryos (Fig. 5H, diagram). Embryos grafted with nuclear-RFP alone formed the well-defined NC streams and migrated normally (Fig. 5H). In contrast, embryos grafted with Piezo1 MO showed NC migration defects (Fig. 5I), displaying the same phenotype observed by *in situ* hybridization in whole embryos and cryosections (Fig. 5A-G). The length of the NC migratory stream was also decreased in Piezo1 MO compared with control grafts (Fig. 5J). We conclude that Piezo1 activity is required specifically within NC cells for normal migration to occur.

### Loss of Piezo1 counteracts semaphorin inhibitory signals

**Fig. 6.**
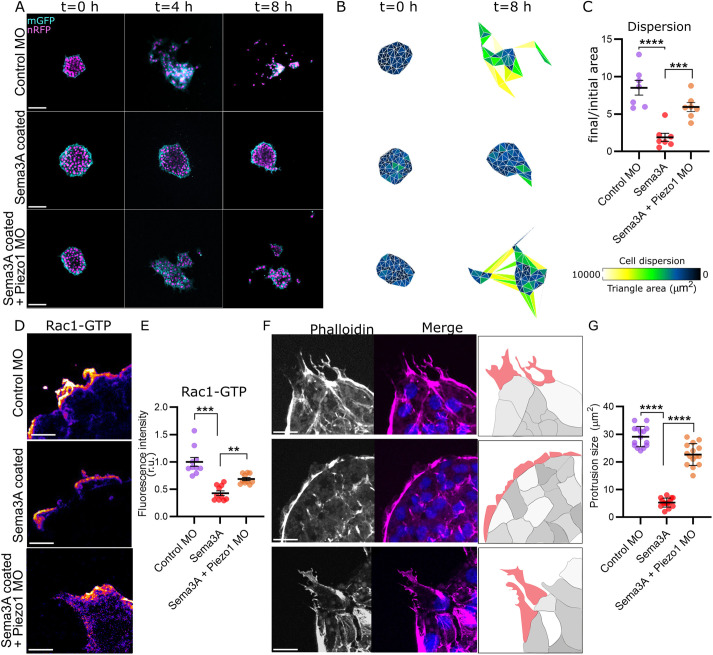
**Piezo1 and Sema3A prevent neural crest invasion via Rac1.** (A) Representative images of NC explants expressing nuclear-RFP (nRFP) and membrane-GFP (mGFP), plated on top of fibronectin (top) or fibronectin plus Sema3A (middle and bottom), at time 0 (left), after 4 h (middle) and after 8 h (right). Note that Piezo1 MO cells disperse in the presence of Sema3A. (B) Analysis of cell dispersion area by Delaunay triangulation, representative areas from A. (C) Normalised area of cell dispersion, showing a ratio of final and initial area from A. *n*=35 explants in each condition. (D) Representative images of NC explants immunostained against Rac1-GTP, plated on top of fibronectin (top) or fibronectin plus Sema3A (middle and bottom). (E) Quantification of fluorescent intensity of Rac1-GTP from D. Rac1 levels are partially rescued in Piezo1 MO. (F) Representative images of cell protrusions from NC explants stained with Phalloidin, plated in the same conditions as D. Note that protrusions are inhibited in explants plated on Sema3A, whereas Piezo1 MO explant protrusions are rescued. Protrusion area is highlighted in red in the illustration. (G) Quantification of protrusion size from F. *n*=10 explants in each condition. Error bars are ±s.e.m. Each dot is the mean value of an independent experiment. All data are representative of at least three biological replicates. **P≤0.01, ***P≤0.001, ****P≤0.0001 (one-way ANOVA with a Dunnett's multiple comparisons post-test). Scale bars: 50 µm (A); 10 µm (D,F).

One of the clearest phenotypes observed upon Piezo1 inhibition is the reduction of the NC gap between streams, which is characteristic of cephalic NC (Fig. 5). In normal NC migration, the gaps between NC streams are generated by inhibitory signals that confine the migratory cells within the streams. One of the best characterized inhibitory signals is Sema, which surrounds the migratory NC (Theveneau and Mayor, 2012). Indeed, inhibition of Sema3A (Bajanca et al., 2019) leads to a phenotype that is very similar to the one observed in Piezo1-depleted cells, described above. Therefore, we hypothesized that in Piezo1 MO embryos, the NC cells circumvent the Sema3A inhibitory signals and migrate into the Sema3A area, thereby losing the stream integrity. To investigate this hypothesis, NC explants were plated on dishes coated with a solution of 60 ng/ml Sema3A, followed by a coating of fibronectin, as previously published (Bajanca et al., 2019). Control NC explants were unable to disperse when plated on Sema3A-coated dishes (Fig. 6A-C; compared with control). In contrast, Piezo1 MO migrated regardless of the Sema3A coating (Fig. 6A-C). Analysis of single cell parameters showed that speed of cell migration is decreased by Sema3A treatment and rescued to control levels in Piezo1 MO plated on Sema3A-coated dishes (Fig. S3D), whereas directionality is not affected (Fig. S3E). The same results were observed when cells were plated on Sema3F (Fig. S3A-E), which inhibits NC migration by the same mechanism as Sema3A. These results indicate that loss of Piezo1 counteracts Sema inhibitory signals in migratory NC.

Sema proteins prevent NC migration by inhibiting Rac1 levels, leading to a collapse of the actin-based protrusions (Bajanca et al., 2019). Given that loss of Piezo1 leads to an increase in active Rac1 levels (Fig. 4F,G), we hypothesised that the mechanism by which Piezo1 MO cells circumvent Sema negative signals involves a misregulation of Rac1 activity. To test this, we analysed Rac1-GTP levels (Fig. 6D). We found a significant decrease in active Rac1 levels on cells plated on Sema3A-coated dishes compared with controls (Fig. 6D,E). However, active Rac1 levels were partially rescued in Piezo1 MO cells plated on top of Sema3A (Fig. 6D,E). Active Rac1 levels were also elevated in Piezo1 MO cells plated on top of Sema3F compared with control cells plated on Sema3F (Fig. S3F,G). Importantly, normal cell protrusions collapse when NC are cultured on Sema3A (Fig. 6F), but they are rescued when Piezo1 is inhibited (Fig. 6F,G). This shows that Piezo1 inhibition counteracts Sema inhibitory signals through Rac1 activity.

### Piezo1 prevents neural crest invasion via Rac1 inhibition

**Fig. 7.**
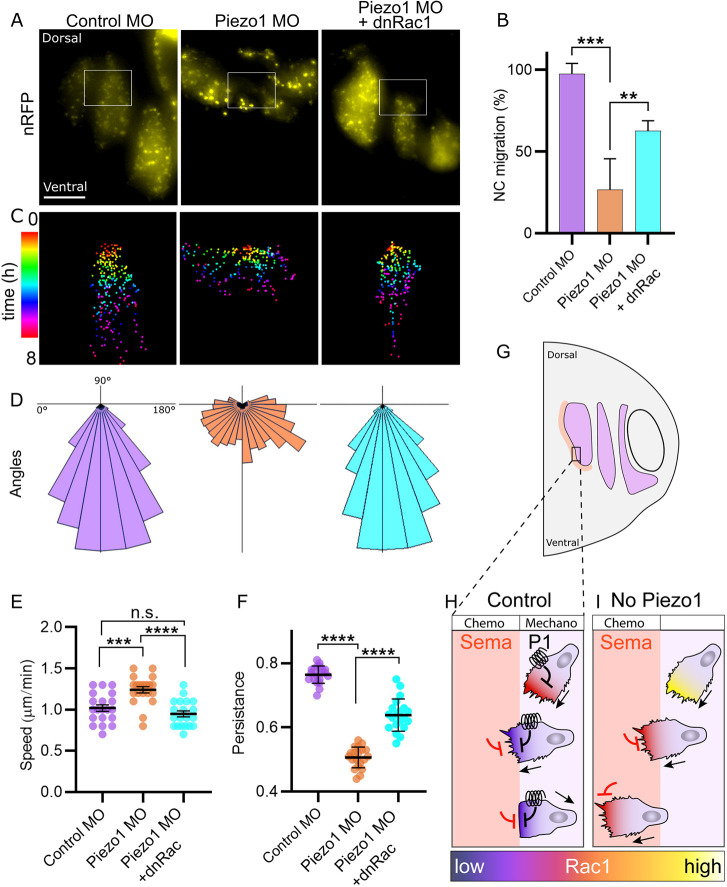
**Piezo1 prevents neural crest invasion via Rac1 inhibition.** (A) NC graft of control (left), Piezo1 MO (middle) and Piezo1 MO plus dominant negative (dn) Rac1 (right). (B) Quantification of the percentage of embryos with normal NC migration from A. Normal NC migration of grafting experiments was determined by comparing the NC migratory streams to a whole-mount *in situ* hybridization. Note that expression of dnRac1 on top of Piezo1 MO partially rescues NC migration. *n*=5 embryos per condition. (C) Colour coded single cell tracks from boxed areas in A. Note that Piezo1 MO cells migrate laterally, whereas directional migration is restored in Piezo1 MO plus dnRac1. (D) Angles of cell migration from C. (E) Quantification of speed of cell migration from A. (F) Quantification of persistence of cell migration from A. Note that both speed and persistence of cell migration are rescued in Piezo1 MO plus dnRac1. *n*=20 in each condition (E,F). Error bars are ±s.e.m. Each dot is the mean value of an independent experiment. All data are representative of at least three biological replicates. **P≤0.01, ***P≤0.001, ****P≤0.0001 (one-way ANOVA with a Dunnett's multiple comparisons post-test). n.s., non-significant. (G-I) Proposed model of the role of Piezo1 (P1) in NC migration. In control embryos (H) Piezo1 mechanical activation partially reduces Rac1 activity, which is further inhibited by chemical signals from Sema. This leads to a collapse of cell protrusions and inhibition of cell migration into the Sema region. In the absence of Piezo1 (I), there is no initial regulation of Rac1 levels: when NC cells reach the Sema region, Rac1 levels are only partially inhibited, and cells continue to invade the Sema region. Scale bar: 100 µm.

The above experiments suggest that the disappearance of the NC stream gaps observed *in vivo* after Piezo1 depletion is due to an increase in Rac1 activity in migrating NC cells that overcomes the inhibition produced by Sema at the stream edges. If this hypothesis is true, partial inhibition of Rac1 in Piezo1 MO embryos would restore the normal Rac1 levels required for cell migration. To test this, we first analysed active Rac1 levels in embryos by immunostaining Rac1-GTP in sagittal cryosections (Fig. S4A). A significant increase in Rac1 was observed in the NC tissue of the Piezo1 MO-injected side compared with the control MO-injected side (Fig. S4B,C). Second, to verify that the effect of Piezo1 on NC migration *in vivo* is Rac1 dependent, we grafted NC from control, Piezo1 MO and Piezo1 MO plus dominant-negative Rac1 (dnRac1) (Habas et al., 2003; Broders-Bondon et al., 2007) into host control embryos (Fig. 7A). As previously observed (Fig. 5), most Piezo1 MO embryos migrated with defects. In contrast, a simultaneous expression of dnRac1 and Piezo1 MO partially rescued the phenotype (Fig. 7A,B).

Detailed analysis of single NC cell behaviour during NC migration *in vivo* was performed. Individual cell tracking of migratory NC cells show that control NC grafts migrate in a directional fashion from dorsal to ventral (Fig. 7C,D), unlike NC explants plated *ex vivo*, which migrate in all directions owing to the lack of attractant or repellent signals (Fig. 2G). Piezo1 MO grafts, however, tend to disperse more, losing the dorsoventral direction and crossing the stream borders (Fig. 7C,D). Strikingly, the directional migration is largely restored when dnRac1 is expressed with the Piezo1 MO (Fig. 7C,D). Analysis of further single cell parameters shows that both speed (Fig. 7E) and persistence (Fig. 7F) of cell migration are rescued in Piezo1 MO plus dnRac1 compared with Piezo1 alone. In conclusion, Piezo1 prevents NC invasion into the Sema region *in vivo* via Rac1 inhibition.

## DISCUSSION

The findings we present here introduce the stretch-activated ion channel, Piezo1, as an important regulator of NC migration. Piezo1 activation in biological systems can only be achieved via mechanical forces applied to the cell plasma membrane (Coste et al., 2010). Therefore, we propose that Piezo1 activity in NC cells is regulated by mechanical stimuli. A potential source for the mechanical cue could be the Sema tissue boundary at the NC stream border, as tissue boundaries have been previously described as sources of mechanical tension (Umetsu et al., 2014). This hypothesis is in accordance with the results presented here, which show that Piezo1 activity is essential at the boundary with Sema proteins to prevent NC invasion. We have previously shown that NC cells can sense and distribute forces through FA proteins *in vivo* (Barriga et al., 2018; Roycroft et al., 2018). The results presented here suggest that the detection and response to mechanical stimuli from NC cells is more complex than previously thought.

The role of Piezo1 on cell migration is controversial, as some reports show that inhibition of Piezo1 increases cell migration, such as in breast cancer and non-small cell lung carcinoma (Huang et al., 2019; Yu et al., 2021), whereas others show inhibition in cell migration, such as in gastric cancer and glioma cells (Chen et al., 2018; Zhang et al., 2018). Our work shows a clear increase in cell motility upon Piezo1 inhibition. In addition, we show that depletion of Piezo1 leads to highly dynamic FA regulation in NC cells, consistent with higher motility. It is interesting to notice that the role of Piezo1 on FA dynamics has been described regardless of whether Piezo1 promotes or inhibits migration in different cell types (Huang et al., 2019; Yu et al., 2021; Chen et al., 2018; Zhang et al., 2018; Chubinskiy-Nadezhdin et al., 2019). These observations indicate that Piezo1 has a conserved role in regulating FA dynamics, but whether this is translated into more or less migration is cell context dependent. The role of Piezo1 in FA dynamics could also be explained by the regulation of calcium levels; it has been shown that inhibition of calcium channels in the NC affect FA and NC migration (Melchionda et al., 2016). Given that calcium, FA and Rac1 are very tightly regulated during cell migration (Matthews et al., 2008; Ciobanasu et al., 2012), one can hypothesise that the extent to which Piezo1 regulates these molecules could lead to inhibition or increase in the speed of cell migration.

Here, we additionally found that Piezo1 alone can affect the speed of cell migration by inducing cytoskeletal remodelling and controlling cell protrusions via the small GTPase Rac1. Interestingly, the mechanism by which Sema3A and Sema3F proteins inhibit NC migration, and control NC stream formation in *Xenopus*, feeds to the regulation of Rac1 (Bajanca et al., 2019). This indicates that both Sema and Piezo1 signals are required for optimal Rac1 levels and NC stream integrity. Given that other inhibitory signals such as ephrins, Slit and Robo act upstream of Rac1 (Riccomagno et al., 2012; Xu and Henkemeyer, 2012; Uemura and Fukushima, 2021), it is likely that Piezo1 cooperates with all the Rac1 inhibitors required for stream formation in a similar fashion to Sema proteins. It is unlikely that Piezo1 affects other types of protrusion formations in our system, as we have previously shown that Cdc42 activity does not change during neural crest migration, as analysed by fluorescence resonance energy transfer (FRET), and this is consistent with the almost absence of filopodia (normally attributed to Cdc42 activity) in *Xenopus* cephalic NC (Matthews et al., 2008).

The role of Piezo1 in NC pattern establishment during migration can be further appreciated in our rescue experiments, where the co-expression of dnRac1 with the Piezo1 MO restores NC stream formation and normal NC migration *in vivo*. Thus, for collective and directional migration *in vivo*, NC cells require simultaneous stimuli from both chemical (Sema) and mechanical (Piezo1) signals. Indeed, Sema KD alone in *Xenopus* embryos can only partially abrogate NC stream formation (Bajanca et al., 2019), which suggests that an additional, previously unknown, mechanism must regulate this process. We now propose Piezo1 as a co-regulator of NC stream patterning formation during cell migration.

Based on what we have identified here, we propose a model for the mechanism by which Piezo1 is involved in NC migration in *Xenopus* in which cells integrate chemical and mechanical signals to control Rac1 in a precise fashion (Fig. 7G). A mechanical stimulus, such as the increase in substrate stiffness that triggers NC migration (Barriga et al., 2018), would activate Piezo1 in NC cells. This activation of Piezo1 would lead to an inhibition of Rac1 activity (Fig. 7H). When the migrating cells reach the border of the NC stream and encounter the Sema area, Rac1 levels would be further inhibited by the chemical signals from Sema, leading to a collapse of the protrusions and inhibition of NC invasion beyond the stream edges (Fig. 7H). However, when Piezo1 is depleted (Fig. 7I), there would be no initial mechanical regulation of Rac1 activity, and when NC cells migrate into the Sema area, Rac1 levels would be only partially inhibited and therefore too high to promote cell protrusion collapse, leading to NC cells continuing to migrate outside their path (Fig. 7I). Our results suggest that the small GTPase Rac1 works as an integrator of mechanical and chemical cues during NC migration. Rac1 regulation is a highly conserved pathway that is essential for mesenchymal cell migration across many different cell types. Therefore, it is likely that the mechanism by which Piezo1 co-operates with Sema signals is conserved in other cell migration and invasion models.

## MATERIALS AND METHODS

### *In vitro* fertilisation and embryo manipulation

Adult *Xenopus laevis* were maintained and used under the regulations and guidelines of the animal licences, which were assigned to this project by the UK Home Office and University College London. *X. laevis* embryos were obtained by *in vitro* fertilization as previously described (Shellard et al., 2018). In brief, ovulation of mature females was induced by human chorionic gonadotrophin (Intervet) injection. Eggs were collected and fertilized *in vitro* by mixing with a sperm solution diluted in 0.1× Marc's Modified Ringer's (MMR) solution. Embryos were maintained in 0.1× MMR at 14°C and staged according to Nieuwkoop and Faber (Grobman, 1958).

For GsMTx4 treatment *in vivo*, embryos were microinjected sub-epidermally at stage 17 (pre-migratory stage), at the cranial neural crest site, with 10 nl of 1 µM GsMTx4 (Abcam, ab141871) in 0.1× MMR. Embryos were incubated until stage 24 (migratory stage) in 1 µM GsMTx4 in 0.1× MMR. Embryos were then fixed in MEMFA and processed by *in situ* hybridization. Control embryos were treated in the same manner as experimental; except that sub-epidermal microinjection and incubation were carried out in 0.1× MMR alone.

### mRNA synthesis, morpholinos, microinjection and reagents

Embryo microinjections were performed with calibrated pulled glass needles according to Shellard et al. (2018). To specifically target the NC, eight-cell-stage embryos were injected into the two right animal ventral blastomeres, the left ventral blastomeres were left non-injected as controls. Fluorescein-dextran (FDx; Invitrogen, D1821, 20 ng) or rhodamine-dextran (RDx; Invitrogen, D1824, 20 ng) were used as tracers when required. Oligomorpholinos against *X. laevis* Piezo1 (5′-CACAGAGGACTTGCAGTTCCATCCC-3′) were designed and synthesized by GeneTools using a previously published sequence (Koser et al., 2016). We then injected 30 ng of Piezo1 MO or scrambled control morpholino (CTLMO: 3′-ATATTTAACATTGACTCCATTCTCC-5′) into each blastomere (total of 60 ng per embryo). The embryos were transferred to a 14°C incubator until the required stage.

mRNA templates were generated as previously described (Shellard et al., 2018). Briefly, mRNA was transcribed with mMESSAGE mMACHINE SP6 Transcription Kit (Thermo Fisher Scientific, AM1340). For cell labelling and tracking, embryos were injected with mRNAs for nuclear RFP (300 pg) and membrane GFP (300 pg). For FA analysis, embryos were injected with FAK-GFP (200 pg). Actin was labelled with LifeAct-Ruby (200 pg). For rescue of *in vivo* migration, embryos were injected with Rac1 N17 (dnRac1; 200 pg). Live movies of GFP-FAK and LifeAct-Ruby were generated using a Leica TCS SP8 confocal microscope with a 63× lens (HCX APO L 63×/0.90 W U-V-I CS2).

### Neural crest dissection, culture and dispersion assay

For *in vitro* experiments, cranial NC was dissected from stage 18 embryos as previously described (Shellard et al., 2018). In brief, the vitelline membrane was carefully removed with tungsten fine forceps. NC was isolated using a hair knife and incubated in 1× Danilchik's for Amy (DFA) medium. For chemical inhibition or activation of Piezo1, GsMTx4 100 nM or Yoda1 20 µM, was added to the DFA medium. Fibronectin coating was carried out by incubating a 10 μg/ml fibronectin solution (Sigma-Aldrich) for 1 h at 37°C. For Sema3A coating, a 60 ng/ml solution, and for Sema3F a 480 ng/ml solution, were incubated for 1 h at 37°C before the fibronectin coating, as previously described (Bajanca et al., 2019). NC explants were plated on top of the coated dishes and allowed to migrate for 8 h while being recorded every 10 min by time-lapse microscopy using a Leica DM5500 compound microscope (Plan Fluor 10×/0.30 DIC L/N1) and a DFC 300FX camera. Data were acquired using LAS acquisition software.

For graft experiments, NC from donor and host embryos were removed in modelling clay, and otherwise as described above. Donor NC was grafted into the location of the removed host NC and a glass coverslip was used to stabilize the grafted NC, as previously described (Barriga et al., 2018). After 1 h, the coverslip was removed, and NC migration was recorded by time-lapse microscopy for 16 h, being recorded every 10 min using a Nikon Eclipse 80i microscope (Plan Fluor 10×/0.30 DIC L/N1) and an ORCA-05G camera (Hamamatsu Photonics). Data were acquired using SimplePCI acquisition software. Normal NC migration of grafting experiments was determined by comparing the NC migratory streams to a wholemount *in situ* hybridization.

### Immunostaining and image analysis

NC cells were cultured on fibronectin-coated coverslips and fixed in 3.7% formaldehyde in PBS for 30 min. Permeabilization was performed with 0.1% Triton X-100 in PBS for 10 min followed by PBS-Tween 0.1% washes. Primary antibodies were incubated for 16 h at 4°C. The following primary antibodies were used: anti-Piezo1 (1:500, ab128245, Abcam); anti-p-paxillin (1:500, pY118, Invitrogen); anti-vinculin (1:300, V9131, Sigma-Aldrich); anti-Rac1-GTP (1:500, sc-514583, Santa Cruz Biotechnology). Alexa fluor Phalloidin (1:500, A12379, Thermo Fisher Scientific); Alexa fluor secondary antibodies (1:500, A11008, A11001, Invitrogen) and Dapi counterstain (20 μg/ml, D9542, Sigma-Aldrich) were incubated for 30 min at 25°C. Imaging was carried out using a Leica TCS SP8 confocal with a 40× lens (HC PL APO 40×/1.30 Oil CS2).

For each experiment, the control and treated samples were manipulated in parallel and following the same staining/imaging conditions. For fluorescence intensity, a background noise subtraction was performed before intensity measurements. The intensity values for each experiment were normalized to the mean of the control.

### *In situ* hybridization

*In situ* hybridization was performed as previously described (Barriga et al., 2018). Briefly, embryos of stage 24 were fixed in MEMFA solution for 1 h, followed by overnight hybridization with a digoxigenin-labelled probe for the migratory NC marker *Twist1* at 0.7 μg/ml (Hopwood et al., 1989). Embryos were then incubated with an anti-digoxigenin antibody (1:2000, 11093274910, Roche) coupled with alkaline phosphatase (AP). AP activity was developed using NBT/BCP substrates.

### TUNEL assay

TUNEL assays were carried out as previously described (Tríbulo et al., 2004). In brief, explants were plated on top of fibronectin as per the dispersion assay and allowed to migrate for 5 h, after which they were fixed in a 4% paraformaldehyde solution. Explants were washed in PBS and incubated in 150 U/ml terminal deoxynucleotidyltransferase (Roche) and 0.5 mM digoxigenin-dUTP (Roche). The reaction was terminated in PBS/1 mM EDTA for 2 h at 65°C, followed by extensive washes in PBS. The explants were then incubated with an anti-digoxigenin antibody coupled to AP at a dilution of 1:2000 (Roche). Explants were washed in PBS and the antibody was visualized using nitroblue tetrazolium and 5-bromo-4-chloro-3-indolyl phosphate as substrates.

The percentage of TUNEL-positive cells was calculated by counting the positively stained nuclei and the total number of cells per explant.

### Calcium imaging

Explants were plated on top of fibronectin as per the dispersion assay. Fluor-8 AM, green fluorescent calcium binding dye (ab142773, Abcam) solution was added to the medium at a final concentration of 10 µM for 15 min, after which fresh medium was added. Imaging was carried out using a Leica TCS SP8 confocal microscope with a 20× lens (HCX APO L 20×/0.50 W U-V-I) at a rate of 10 images per second. Fluorescence intensity was measured in all time frames as previously described in the Immunostaining and image analysis section.

### Cryosections

Cryosections were performed as previously described (Barriga et al., 2018). Fixed embryos were washed twice for 5 min with phosphate buffer (PB; 0.2 M NaH2PO4*H2O and 0.2 M K2HPO4, pH 7.4), incubated for 2 h at room temperature with a solution containing 15% sucrose (Sigma-Aldrich) in PB (w/v) and 1 h at 42°C in a gelatin solution, containing 8% gelatin (Sigma-Aldrich) and 15% sucrose in PB (w/v). Embryos were oriented in gelatin solution and gelatin blocks containing the embryos were snap frozen at −80°C with pre-chilled isopentane. Samples were sectioned in 20 μm slices using a cryostat (CM-3050S, Leica) and collected in SuperFrozen^®^ Slides (VWR International). The slides were dried for at least 6 h and the gelatin was removed by washing twice with PBS for 15 min. Sections were treated following the immunostaining protocol.

### Analysis of neural crest migration

Analysis of NC dispersion was carried out as previously described (Carmona-Fontaine et al., 2011). In brief, an ImageJ custom-made Delaunay triangulation plugin was used to calculate the area between neighbour cells. To measure speed and directionality of cell migration, cells were tracked manually using the ManualTracker ImageJ plugin; individual cells within an explant were tracked at 10 min intervals for 8 h, the average of all cells analysed per explant is reported in the respective figures.

FA were identified at the substrate focal plane. Length and area measurements were carried out at specific regions of interest of FA accumulation at the basal side of the cells.

Protrusions were defined as the new membrane generated between consecutive frames in a time-lapse movie of NC cells labelled with membrane-GFP as previously described (Matthews et al., 2008). Briefly, the Image Calculator tool on ImageJ was used to subtract membrane images from consecutive frames during time-lapse microscopy.

### Statistical analysis

Statistical analyses were carried out using Prism9 (GraphPad). The types of statistical tests and exact value of *n* (sample size) are mentioned for each experiment in the corresponding figure legend. D'Agostino's K-squared test was used to assess normality of the datasets. Unpaired two-tailed Student's *t*-test was used for samples of normal distribution. For significance we used the convention: ****P≤0.0001, ***P≤0.001, **P≤0.01, *P≤0.05, n.s., non-significant.

## Supplementary Material

Supplementary information
