# Supplementary material for: The mechanosensitive channel Piezo1 cooperates with semaphorins to control neural crest migration
Source: Development. 2021 Dec 2;148(23):dev200001. doi: 10.1242/dev.200001 (PMC8714065; doi:10.1242/dev.200001)
Supplement: Supplementary information [file develop-148-200001-s1.pdf]

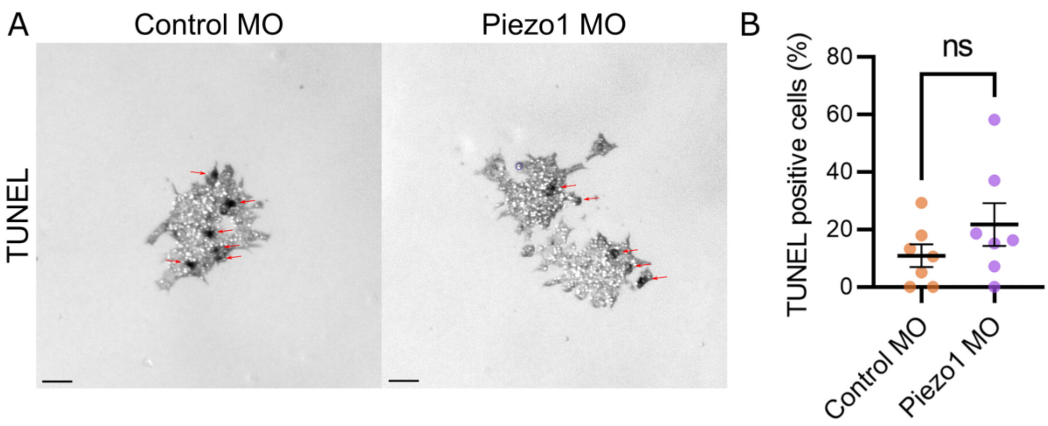

Fig. S1. Piezo1 knock down has no effect on apoptosis. (A) Representative images of NC explants plated on top of fibronectin and fixed with paraformaldehyde. Explants were labelled by TUNEL staining. Red arrows point to TUNEL positive cells. **(B)** Quantification of percentage of TUNEL positive cells in migratory neural crest explants. No significant difference was found between control MO and Piezo1 MO cells. (n=140) cells per condition. Scale bars, 20 µm. Error bars are ±SEM. Each dot is the mean value of an independent experiment. All data is representative of at least 3 biological replicates. Student's *t*-test (two-tailed). n.s. non-significant.

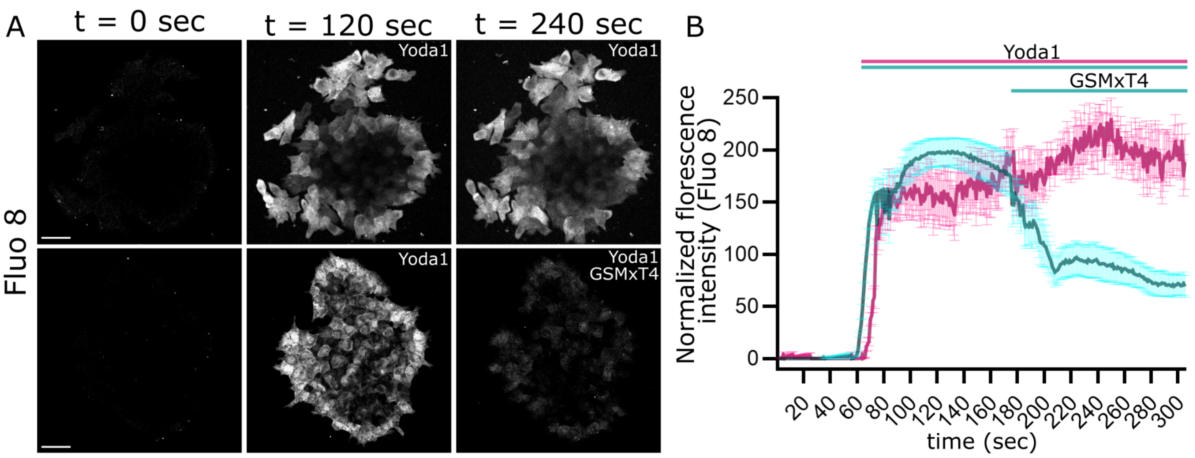

Fig. S2. Reversibility of Piezo1 inhibition. (A) Representative images of NC explants plated on top of fibronectin and treated with Fluor8, calcium fluorescent reporter dye. Top panel shows a neural crest explant before and after treatment with the Piezo1 activator, Yoda1. Note that Yoda1 induces Fluor8 signal that is sustained after 240 sec. Bottom panel shows a neural crest explant before and after treatment with Yoda1, followed by addition of the Piezo1 inhibitor GSMxT4. Note that Fluor8 signal is decreased after the addition of GSMxT4 at 240 sec. **(B)** Quantification of the Fluor 8 fluorescence levels from (A). n=50 cells. Scale bars, 20 µm. Error bars are ±SEM. All data is representative of at least 3 biological replicates.

Development • Supplementary information

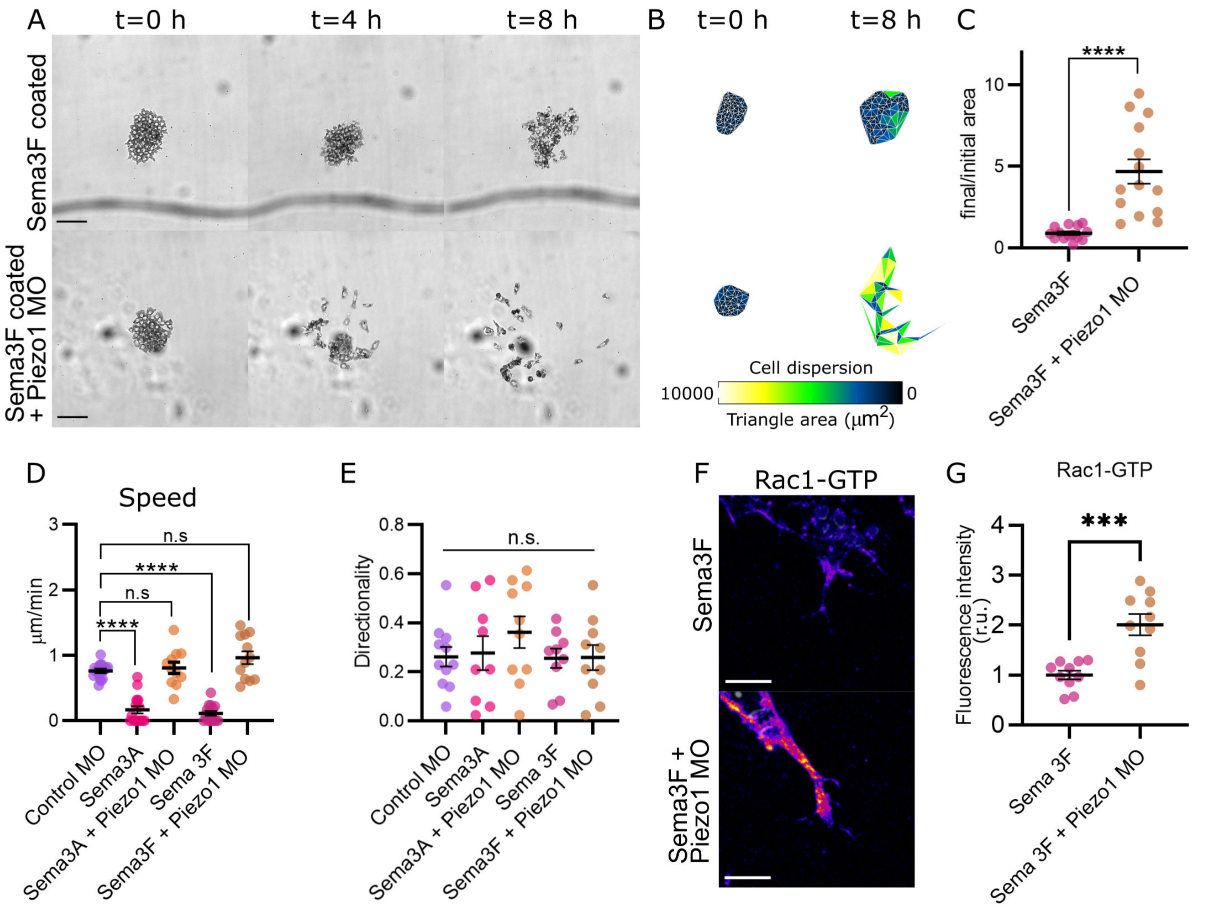

Fig. S3. Loss of Piezo1 counteracts Sema3F and Sema3A inhibitory signals. (A) Representative images of NC explants plated on top of fibronectin plus Sema3F, at time 0 (left column), after 4 hours (middle column) and after 8 hours (right column). Note that Piezo1 MO cells disperse in the presence of Sema3F. **(B)** Analysis of cell dispersion area by Delaunay triangulation, representative areas from (A). **(C)** Normalised area of cell dispersion, showing a ratio of final and initial area from (A). n=14 explants in each condition. **(D)** Quantification of speed of cell migration from (A and Fig 6A). **(E)** Quantification of directionality of cell migration from (A and Fig 6A). (E,G); n=50 cells per condition. **(F)** Representative images of NC explants immunostained against Rac1-GTP, plated on top of fibronectin plus Sema3F. **(G)** Quantification of fluorescent intensity of Rac1-GTP from (F). Rac1 levels are partially rescued in Piezo1 MO. n=10 explants in each condition. (A) Scale bars=50μm. (F) Scale bars=10μm. Error bars are ±SEM. Each dot is the mean value of an independent experiment. All data is representative of at least 3 biological replicates. (C,G) Student's *t*-test (two-tailed). (D-E) One-way ANOVA with a Dunnett's multiple comparisons post-test. ***$p \leq 0.001$, ****$p \leq 0.0001$, n.s. non-significant.

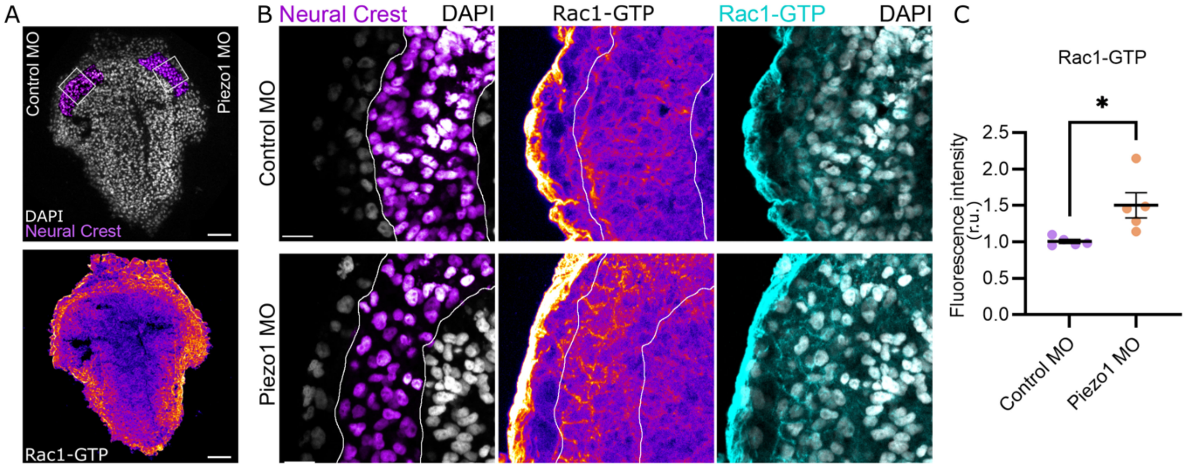

Fig. S4. Increased active Rac1 upon Piezo1 knock down *in vivo*. (A) Sagittal cryosections of whole embryos injected on the left side with control MO and on the right side with Piezo1 MO. Top. Nuclei are labelled with DAPI, neural crest is indicated in purple. Bottom. Rac1-GTP immunostaining labels active Rac1. Note that there is increased Rac1-GTP signal on the Piezo1 MO injected side (right side). Scale bars=100µm **(B)** Higher magnification images of neural crest tissue from (A), each condition is indicated in the figure. Scale bars=20µm. **(C)** Quantification of Rac1-GTP levels of neural crest cells from (B). n=50 cells. Error bars are ±SEM. Each dot is the mean value of an independent experiment. All data is representative of at least 3 biological replicates. Student's *t*-test (two-tailed). * $p \leq 0.05$.

Development • Supplementary information

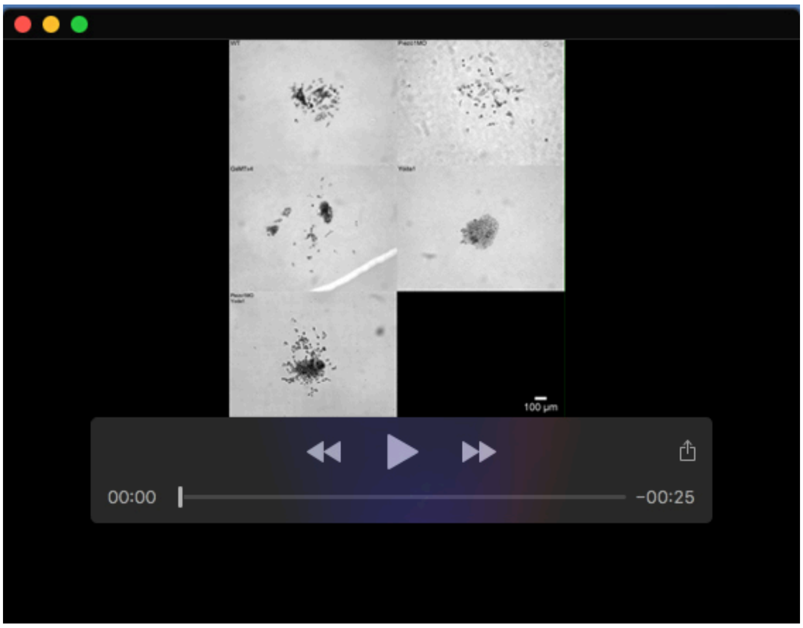

Movie 1. Piezo1 inhibition increases speed of neural crest cell dispersion. Time lapse videos of neural crest cultured on fibronectin with the indicated treatments. 10X magnification. Total length: 8h.

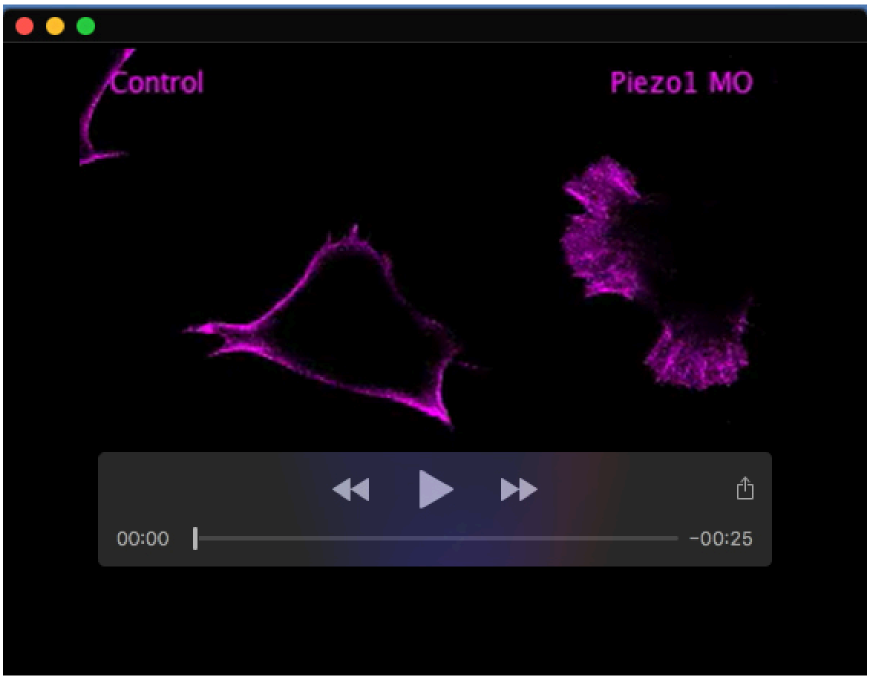

Movie 2. Loss of Piezo1 leads to increased protrusion activity. Time lapse of neural crest cells plated on a fibronectin coated dish, expressing LifeAct-Ruby in control MO and Piezo1 MO. 40X magnification. Total length: 30min.
